# Telomere Length in Peripheral Blood Leukocytes Is Associated with Risk of Colorectal Cancer in Chinese Population

**DOI:** 10.1371/journal.pone.0088135

**Published:** 2014-02-03

**Authors:** Qin Qin, Jingwen Sun, Jieyun Yin, Li Liu, Jigui Chen, Yuxing Zhang, TingTing Li, Yun Shi, Sheng Wei, Shaofa Nie

**Affiliations:** 1 Department of Epidemiology and Biostatistics, and the Ministry of Education Key Lab of Environment and Health, School of Public Health, Tongji Medical College, Huazhong University of Science and Technology, Wuhan, Hubei, China; 2 The Chengdu Centers for Disease Control and Prevention, Chengdu, Sichuan, China; 3 Department of Surgery, The Eighth Hospital of Wuhan, Wuhan, Hubei, China; Centro di Riferimento Oncologico, IRCCS National Cancer Institute, Italy

## Abstract

**Background:**

Human telomeres, tandem repeats of TTAGGG nucleotides at the ends of chromosomes, are essential for maintaining genomic integrity and stability. Results of previous epidemiologic studies about the association of telomere length with risk of colorectal cancer (CRC) have been conflicting.

**Methods:**

A case-control study was conducted in a Han population in Wuhan, central China. The relative telomere length (RTL) was measured in peripheral blood leukocytes (PBLs) using quantitative real-time polymerase chain reaction (PCR) in 628 CRC cases and 1,256 age and sex frequency matched cancer-free controls. Odds ratios (OR) and 95% confidence intervals (95% CI) were calculated using unconditional logistic regression models to evaluate the association between RTL and CRC risk.

**Results:**

Using median RTL in the controls as the cutoff, individuals with shorter RTL were associated with a significantly increased risk of CRC (adjusted OR = 1.27, 95%CI: 1.05–1.55). When participants were further categorized into 3 and 4 groups according to the tertile and quartile RTL values of controls, significant relationships were still observed between shorter RTL and increased CRC risk (OR per tertile = 1.13, 95%CI: 1.00–1.28, *P*_trend_ = 0.045; OR per quartile = 1.12, 95%CI: 1.03–1.23, *P*_trend_ = 0.012). In stratified analyses, significant association between shorter RTL and increased CRC risk was found in females, individuals younger than 60 years old, never smokers and never drinkers.

**Conclusions:**

This study suggested that short telomere length in PBLs was significantly associated with an increased risk of CRC in Chinese Han population. Further validation in large prospective studies and investigation of the biologic mechanisms are warranted.

## Introduction

Human telomeres are tandem repeats of TTAGGG nucleotides that cap the ends of the eukaryotic chromosome arms[1], [2]. Telomeres are folded into loop structures and play important role in maintaining genomic structural integrity and stability by preventing fatal incidents such as nucleolytic degradation, chromosome end-to-end fusion and irregular recombination[3], [4]. In normal somatic cells, human telomeres are approximately 10–15 kb and progressively shortened by 30 to 200 base pairs after each cycle of mitotic division, due to the“end replication problem” and the absence of a mechanism for elongation of telomeres[5]–[7]. Previous reports have indicated that, in addition to the mitotic replication rate, many endogenous and exogenous risk factors such as oxidative stress, smoking, obesity and low socioeconomic status may also contribute to the rate of telomere attrition[8]–[12].

When telomeres shorten to a critical length, they become dysfunctional, and *Rb* and/or *p53* signal pathways will be triggered to initiate cellular senescence or apoptosis[13], [14]. If apoptosis does not happen and cell division continues, the resultant genomic instability will lead to chromosomal abnormalities. Somatic cancer cells, lack of normal DNA damage response mechanisms, continue to divide despite critically short telomeres by utilizing the alternative telomeres prolongation mechanism or upregulating of telomerase[15], [16]. Genome instability is a hallmark of tumorigenesis and is a wildly accepted view as a major contribution to the development of cancer[17], [18].

Several epidemiological studies have evaluated the relationship between telomere length and risk of cancers, but the results are inconsistent. Some investigations have demonstrated that shorter telomere length in PBLs is associated with increased risk of several cancers including lung, bladder, gastric, esophageal, ovarian, head and neck and renal cancer[10], [19]–[25]. In contrast, some reports have suggested that longer telomere length may be associated with increased risk of melanoma and breast cancer, non-Hodgkin lymphoma and soft tissue sarcoma[26]–[29]. Mouse models and functional studies support that telomere shortening is involved in initiation, development and progression of malignancies[30], [31]. For example, short telomeres lead to an increased risk of developing epithelial cancers by the formation of complex non-reciprocal translocations [32], and telomeres in tumor tissues and their precursor lesions are significantly shorter than that in adjacent non-tumor tissues [33], [34].

About CRC, results from previous studies have been conflicting. Three studies[35]–[37] showed null associations between telomere length and CRC risk. One study[36] reported shorter telomeres associated with increased CRC risk. One recent study[38] found longer telomeres associated with increased CRC risk among carriers of the common allele at a SNP near *TERC*. Studies above were all restricted to Europeans. Cui and colleagues[39] observed a U-shaped association between telomere length and CRC risk among Chinese women, which suggested that both very short and very long telomeres were associated with increased risk of CRC. However, such finding has not been verified by later studies. To further investigate the association between telomere length and CRC risk, we conducted a case-control study in a Chinese Han population.

## Materials and Methods

### Study population

The study population consisted of 628 newly diagnosed CRC cases and 1256 cancer-free controls. Subject recruitment and data and specimen collection methods have been described previously[40]. Briefly, between January 1, 2007 and December 31, 2010, patients were consecutively recruited at the Eighth Hospital of Wuhan, Wuhan City, central China. Controls were cancer-free individuals living in Wuhan city and surrounding regions, randomly selected from the health examination population in the same hospital during the same period as the cases were enrolled. The inclusion criteria for patients included histopathologically confirmed CRC, without previous radiotherapy or chemotherapy and no restriction in regards to age, sex or disease stage. Cases with pathology report designated Lynch syndrome, colorectal adenoma, inflammatory bowel disease, familial adenomatous polyposis, schistosomiasis and intestinal tuberculosis were excluded. The selection criteria for controls included cancer-free individuals and frequency matched to cases by sex and age (±5 years). All subjects were unrelated ethnic Han Chinese. Demographic and lifestyle information and medical data were collected by trained interviewers via direct interview using questionnaires. Clinical data were abstracted from hospital medical records. 5-ml peripheral venous blood was drawn from each participant. All participants provided written informed consent at enrollment. The study was approved by the institutional review board of School of Public Health of Tongji Medical College of Huazhong University of Science and Technology.

### Telomere length determination

Genomic DNA was isolated from participants' PBLs using the Relax Gene Blood DNA System DP319-02 (Tiangen, Beijing, China) according to the manufacturer's protocol. The RTL of each DNA sample was measured using a unified real-time quantitative PCR protocol originally described by Cawthon[41] with minor modifications. In brief, two master mixes of PCR reagents were prepared: one for telomere reaction and one for a human single-copy gene reaction (*36B4* on chromosome 12). The real-time PCR was conducted on an ABI 7900HT Sequence Detection System (Applied Biosystems). The PCR reaction mixture (10 µL) for the telomere or *36B4* amplification consisted of 1 X SYBR Green Master Mix (Toyobo), 300 nmol/L each telomere or *36B4* specific primers, and 5 ng genomic DNA. The thermal cycling conditions for both telomere and *36B4* were 95°C for 10 minutes to activate Taq-polymerase followed by 40 cycles of denaturation at 95°C for 15 seconds and annealing-extension at 60°C for 1 minute. The primer sequences for both PCR reactions have been previously published[41]. All samples for both the telomere and single-copy gene amplifications were done in duplicate on the same 384-well plate. Melting (dissociation) curve analysis was performed on every run to verify specificity and identity of the PCR products. Standard curve for RTL measurement was constructed on each plate with a reference DNA sample. The reference DNA was the pooled genomic DNA of 50 randomly selected controls. For each standard curve, the reference DNA sample was diluted by using a 2-fold serial dilution to generate a 6-point standard curve, between 20 and 0.625 ng DNA in each reaction. Every standard curve point was run in triplicate. All plates in this study used the same reference DNA. The purpose of the standard curve was to assess and compensate for inter-plate variations in PCR efficiency. The ratio of telomere repeat copy number to the single-copy gene copy number (T/S), characterizing RTL, was determined for each sample based on the standard curve. Laboratory workers were blinded to each sample's case-control status. The acceptable standard deviation was set at 0.3 for the threshold cycle (*C*_t_) values and the R^2^ correlation for each standard curve was ≥0.98. If the result was out of the acceptable range, then the run was repeated for the same sample. For testing intra- and inter-plate variation, the coefficients of variation (CVs) for the T/S ratio of the reference DNA at the 5 ng point were calculated. And the intra- and inter-plate CVs were 4.6% and 8.9%, respectively.

### Statistical analysis

The differences in the distribution of baseline characteristics between cases and controls were compared using the chi-square test for categorical variables, and the Student's *t*-test for continuous variables. RTL data were natural log-transformed so that these data were approximately normally distributed. RTL was analyzed as categorical variable based on the cut-off points at the median, tertile, and quartile value among controls. ORs and 95% CIs were calculated using unconditional logistic regression models to estimate the association between RTL and CRC risk. By treating the level of telomere length as a continuous variable, tests for trend were calculated. Adjusted analyses included terms for age, sex, smoking status and alcohol use. Stratified analyses were conducted to evaluate potential interactions between demographic factors and RTL on CRC risk. The power of our sample size was calculated to be 0.87 to detect a trend in ORs with decreasing quartiles of RTL assuming an OR of 1.4 comparing the lowest and highest quartiles, at an alpha of 0.05. All statistical analyses were performed using SPSS software 12.0 (SPSS, Inc., Chicago, III) and all *P* values were tested two-tailed with a significant level at 0.05.

## Results

The baseline characteristics of the study participants were shown in Table 1. There were no significant differences in the distribution of age, sex and body mass index (BMI) between cases and controls. The mean age [± standard deviation (SD)] was 58.8 (±11.8) years old for cases and 58.8 (±11.4) years old for controls (*P* = 0.952). More smokers and drinkers were observed in cases than in controls (*P*<0.001).

**Table 1:** Baseline characteristics of study participants

	Cases	Controls	
Characteristics	No. (%)	No. (%)	*P*
Total	628	1256	
Age (years)			0.952^a^
(Mean±SD)	58.8±11.8	58.8±11.4	
Sex			0.768^b^
Female	288(45.9)	567(45.1)	
Male	340(54.1)	689(54.9)	
Smoking status			3.192×10^−5b^
Never	388(61.8)	895(71.3)	
Ever	240(38.2)	361(28.7)	
Alcohol use			3.573×10^−4b^
Never	449(71.5)	991(78.9)	
Ever	179(28.5)	265(21.1)	
BMI (kg/m^2^)			0.175^a^
(Mean±SD)	23.23±3.34	22.99±3.20	

Abbreviations: SD, standard deviation; BMI, body mass index.

a Caculated by Student's *t*-test;

b Caculated by Pearson's *χ^2^* test.

We estimated the association between RTL and the risk of CRC by unconditional logistic regression analysis, through treating RTL as a categorical variable based on a cut off value of median, tertile and quartile distribution in controls. As shown in Table 2, individuals with shorter RTL by median cut-off had a significantly increased risk of CRC (adjusted OR = 1.27, 95%CI: 1.05–1.55). When participants were categorized into 3 groups according to tertile values of RTL in controls, we observed a borderline significant relationship between shorter RTL and increased CRC risk (OR per tertile = 1.13, 95%CI: 1.00–1.28, *P*_trend_ = 0.045). When the third (longest) tertile was used as the reference group, the adjusted OR for the second and first group were 1.66 (95%CI: 1.30–2.11) and 1.32 (95%CI: 1.03–1.69), respectively. Similarly, when participants were further categorized into 4 groups according to quartile values of RTL in controls, we also observed a significant relationship (OR per quartile = 1.12, 95%CI: 1.03–1.23, *P*_trend_ = 0.012). When the fourth (longest) quartile was used as the reference group, the adjusted ORs for the third, second, and first quartiles were 1.77 (95%CI: 1.32–2.38), 2.05 (95% CI: 1.54–2.74), and 1.47 (95%CI: 1.09–1.99), respectively.

**Table 2:** Association between telomere length and colorectal cancer risk in a Chinese population.

Telomere length	Cases	Controls	Crude OR (95%CI)^a^	*P* a	Adjusted OR (95%CI)^b^	*P* b
By median						
Long	275	628	1.00		1.00	
Short	353	628	1.28(1.06–1.56)	0.011	1.27(1.05–1.55)	0.015
By tertile						
3rd tertile	156	418	1.00		1.00	
2nd tertile	264	420	1.68(1.33–2.14)	2.039×10^−5^	1.66(1.30–2.11)	4.209×10^−5^
1st tertile	208	418	1.33(1.04–1.71)	0.023	1.32(1.03–1.69)	0.031
Per tertile			1.14(1.01–1.29)	*P*_trend_ = 0.034	1.13(1.00–1.28)	*P*_trend_ = 0.045
By quartile						
4th quartile	99	314	1.00		1.00	
3rd quartile	176	314	1.78(1.33–2.38)	1.107×10^−4^	1.77(1.32–2.38)	1.461×10^−4^
2nd quartile	206	314	2.08(1.56–2.77)	5.225×10^−7^	2.05(1.54–2.74)	9.973×10^−7^
1st quartile	147	314	1.49(1.10–2.00)	0.010	1.47(1.09–1.99)	0.012
Per quartile			1.13(1.03–1.23)	*P*_trend_ = 0.009	1.12(1.03–1.23)	*P*_trend_ = 0.012

Abbreviations: OR, odds ratio; CI, confidence interval.

a Crude results without adjustment.

b Adjusted by age, sex, smoking status and alcohol use.

Stratified analyses were conducted to evaluate the potential modulating effect of each host variables. We found a significant association between shorter RTL and increased CRC risk in females (OR = 1.72, 95% CI: 1.28–2.31) and individuals younger than 60 years old (OR = 1.39, 95% CI: 1.06–1.83), but not in males and participants older than 60 years old (Table 3). Significant associations were also observed in never smokers (OR = 1.52, 95% CI: 1.19–1.93) and never drinkers (OR = 1.40, 95% CI: 1.12–1.75).

**Table 3:** Association of telomere length with colorectal cancer risk stratified by selected characteristics

Characteristic	TL(by median)	Cases	Controls	OR (95%CI)^a^	*P* a
Age					
<60	Long	143	315	1.00	
	Short	189	306	1.39(1.06–1.83)	0.017
> = 60	Long	132	313	1.00	
	Short	164	322	1.17(0.88–1.55)	0.277
Sex					
Female	Long	111	292	1.00	
	Short	177	275	1.72(1.28–2.31)	2.979×10^−4^
Male	Long	164	336	1.00	
	Short	176	353	1.02(0.78–1.33)	0.892
Smoking status					
Never	Long	159	460	1.00	
	Short	229	435	1.52(1.19–1.93)	0.001
Ever	Long	116	168	1.00	
	Short	124	193	0.93(0.67–1.30)	0.678
Alcohol use					
Never	Long	190	503	1.00	
	Short	259	488	1.40(1.12–1.75)	0.004
Ever	Long	85	125	1.00	
	Short	94	140	0.98(0.67–1.45)	0.927

Abbreviations: TL, telomere length; OR, odds ratio; CI, confidence interval.

a Adjusted by age, sex, smoking status and alcohol use.

## Discussion

In the current study, we investigated the association between telomere length in PBLs and the risk of CRC. We found that short telomere length was significantly associated with an increased risk of CRC in Chinese Han population. Further stratified analyses revealed that the association was significant in women, individuals younger than 60 years old, never smokers and never drinkers.

Mixed findings have been identified from the numerous studies that have extensively investigated the relationship between altered telomere length and cancer risk, and the association appears to be cancer type-dependent[10], [19]–[23], [26]–[29]. Our current study, demonstrating that short telomere length in PBLs was associated with an increased risk of CRC, is consistent with the majority of previous reports, which were combined in two meta-analyses[24], [42]. The first[42] reported a pooled OR of 1.69 (95% CI: 1.53–1.87) for cancers of the digestive system. The second meta-analysis[24] reported pooled ORs of cancer for individuals with the shortest telomeres vs. the longest of 1.16 (95% CI: 0.87–1.54) in prospective studies and of 2.90 (95% CI: 1.75–4.80) in retrospective studies.

About the association between leukocyte telomere length and CRC risk, results of previous studies were conflicting. Pooley and colleagues[36] found a strong association between short telomere length and CRC risk in the retrospective study using data from the SEARCH colorectal cancer case-control study, but not in the prospective study with data from the EPIC-Norfolk cohort. Another two relatively small studies[35], [37] about the relationship between telomere length and CRC risk showed no association. Both studies were respectively restricted to European males and females from large cohorts (Women's Health Study and Physician's Health Study). However, a recent large study[38] with European samples, found that carriers of the common allele at a SNP near the TERC had significantly longer telomeres in leukocytes and found the same allele to be associated with increased CRC risk. Among Chinese women, Cui and colleagues[39] found a U-shaped association between telomere length and CRC risk, indicating both very short and very long telomeres to be associated with increased risk of CRC. Our finding was consistent with the result of Pooley and colleagues' retrospective study. In this study, we did not find the U-shaped association between telomere length and CRC risk, neither among women or men. Several potential reasons may account for these discrepancies, e.g., the study population (ethnicity, sex, age, lifestyle factors), sample size, study design, telomere length measurement and analytical method used. Pooley and colleagues considered that telomere length difference observed could be an effect of cancer treatment and it was possible that telomere attrition was a response to a particular aspect of treatment or treatment regime. But in our study the cases were newly diagnosed without previous chemotherapy or radiotherapy. Alternatively, changes in telomere length may occur systemically during disease development.

Significant association between short telomere length and increased CRC risk observed in our study is biologically plausible. Various experiments and genetic studies support such a hypothesis that telomere attrition leads to the manifestation and dissemination of malignancies[43]. Although fully functional telomeres protect the genome, shortened telomeres cause chromosomal instability[5]. Through the balance of cell proliferation, senescence and apoptosis, optimal telomere length is achieved[44]. In a mouse model, the lack of the telomerase RNA component caused a markedly increased frequency of chromosomal aberration and sporadic cancer[45], especially in the case of simultaneous inactivation of the tumor suppressor gene *p53* [32]. It is reasonable to suppose that cells with critically short telomere length may in some cases reactivate the enzyme telomerase to bypass cell senescence and thereby promote malignant transformation. T lymphocytes upregulate *TERT* on activation by inflammatory stimuli potentially linked to short telomere length[46].

Some limitations in this study need to be addressed. First, the sample size of our study for some stratified analyses remains relatively small, resulting in the relatively inadequate statistical power. Second, although we only included newly diagnosed CRC patients before any treatments, which may reduce the possible influence of disease status and treatment on telomere length, we could not completely get over the inherited limitation of “reverse causation” in a retrospective case-control study. Third, telomere length was only measured in leukocytes from peripheral blood and not in colorectal tumor tissues; however, leukocyte telomere length has been demonstrated to be correlating highly with that in cells from other tissues[47]–[49].

In conclusion, this study suggested that short telomere length in PBLs was significantly associated with an increased risk of CRC in Chinese Han population. Further validation in large prospective studies and investigation of the biologic mechanisms are warranted.
